# Phenylethanolamine N-methyltransferase downregulation is associated with malignant pheochromocytoma/paraganglioma

**DOI:** 10.18632/oncotarget.8234

**Published:** 2016-03-21

**Authors:** Seung Eun Lee, Ensel Oh, Boram Lee, Yu Jin Kim, Doo-Yi Oh, Kyungsoo Jung, Jong-Sun Choi, Junghan Kim, Sung Joo Kim, Jung Wook Yang, Jungsuk An, Young Lyun Oh, Yoon-La Choi

**Affiliations:** ^1^ Department of Pathology, Konkuk University School of Medicine, Konkuk University Medical Center, Seoul, Korea; ^2^ Department of Pathology and Translational Genomics, Samsung Medical Center, Sungkyunkwan University School of Medicine, Seoul, Korea; ^3^ Laboratory of Cancer Genomics and Molecular Pathology, Samsung Biomedical Research Institute, Samsung Medical Center, Sungkyunkwan University School of Medicine, Seoul, Korea; ^4^ Department of Health Sciences and Technology, SAIHST, Sungkyunkwan University, Seoul, Korea; ^5^ The Center for Anti-Cancer Companion Diagnostics, School of Biological Science, Institutes of Entrepreneurial BioConvergence, Seoul National University, Seoul, Korea; ^6^ Department of Surgery, Samsung Medical Center, Sungkyunkwan University School of Medicine, Seoul, Korea; ^7^ Department of Pathology, Gyeongsang National University School of Medicine, Jinju, Korea; ^8^ Department of Pathology, Gachon University Gil Medical Center, Incheon, Korea

**Keywords:** pheochromocytoma/paraganglioma, phenylethanolamine N-methyltransferase, biomarker, metastasis, endocrine tumors

## Abstract

Malignant pheochromocytoma/paraganglioma (PCC/PGL) is defined by the presence of metastases at non-chromaffin sites, which makes it difficult to prospectively diagnose malignancy. Here, we performed array CGH (aCGH) and paired gene expression profiling of fresh, frozen PCC/PGL samples (*n* = 12), including three malignant tumors, to identify genes that distinguish benign from malignant tumors. Most PCC/PGL cases showed few copy number aberrations, regardless of malignancy status, but mRNA analysis revealed that 390 genes were differentially expressed in benign and malignant tumors. Expression of the enzyme, phenylethanolamine N-methyltransferase (PNMT), which catalyzes the methylation of norepinephrine to epinephrine, was significantly lower in malignant PCC/PGL as compared to benign samples. In 62 additional samples, we confirmed that PNMT mRNA and protein levels were decreased in malignant PCC/PGL using quantitative real-time polymerase chain reaction and immunohistochemistry. The present study demonstrates that PNMT downregulation correlates with malignancy in PCC/PGL and identifies *PNMT* as one of the most differentially expressed genes between malignant and benign tumors.

## INTRODUCTION

Pheochromocytoma/paraganglioma (PCC/PGL) is defined by the presence of metastases at non-chromaffin sites distant from the primary tumor, such as lung, liver, bone and lymph nodes, rather than local invasion [1]. Although PCC/PGL malignancy rate varies with tumor site and the particular inherited mutation in familial diseases, it is difficult to predict malignancy in primary tumors [2, 3]. Therefore, biomarkers that can indicate subsets of tumors that may metastasize must be identified.

Establishment of histopathological malignancy criteria is challenging due to the rarity, heterogeneity and lengthy tumor development time of PCL/PGL. In 2002, Thompson developed the Pheochromocytoma of the Adrenal Gland Scaled Score (PASS) system, consisting of 12 parameters weighted according to relative frequency in benign and malignant adrenal PCC/PGLs [4]. Tumors with a PASS ≥ 4 were identified as potentially aggressive. Although this scoring system has been validated independently, there is debate as to its utility and reproducibility [5, 6]. Consequently, there are no consistently reliable histological features to distinguish malignant from benign tumors or to predict malignancy using resected primary tumor tissue.

Several attempts have been made to establish useful molecular markers for PCC/PGL malignancy. Mutations of the succinate dehydrogenase subunit B gene (*SDHB*) were associated with high rates of malignancy and extra-adrenal tumor metastasis [7, 8]. Non-diploid tumors were also found to be associated with malignancy [9]. Comparative genomic hybridization (CGH) studies in sporadic PCC/PGL revealed loss of chromosomes 1p, 3q, and 6q and gain of 9q, 16p, 17q, 19p and 19q [10, 11]. One study reported that progression to malignancy was strongly associated with deletions on chromosomes 6q and 17p [10]. Another revealed that alterations of chromosome 11, especially loss of 11q22–23, were more frequent in malignant than benign tumors [11]. In a gene expression profile study, over 80% of differentially expressed genes were downregulated in malignant tumors [17]. In contrast, HSP90 [18], human telomerase reverse transcriptase (hTERT) [19], tenascin [20], N-cadherin [21] and COX-2 [20] were overexpressed in malignant tumors.

Here, we performed array CGH (aCGH) and paired gene expression profiling of fresh, frozen PCC/PGL samples (*n* = 12), including three malignant tumors, to identify genes that distinguish benign from malignant tumors. Integrated analysis of the data identified phenylethanolamine-N-methyltransferase (*PNMT*) as a candidate marker. This was validated using real-time quantitative reverse-transcriptase polymerase chain reaction (qRT-PCR) and immunohistochemistry (IHC) in an independent set of 62 PCC/PGL samples.

## RESULTS

### Patient clinicopathological characteristics

Fresh, frozen PCC/PGL specimens were obtained from nine patients with benign tumors and three patients with malignant tumors, classified based on the absence or presence of metastatic lesions. Detailed clinicopathological characteristics of these 12 PCC/PGL patients are presented in Table 1. Additionally, 62 formalin-fixed paraffin-embedded (FFPE) PCC/PGL samples were analyzed, including 19 malignant and 31 benign specimens. Benign and malignant PCC/PGL patient clinicopathological characteristics were compared (Table 2). All patients presented non-syndromic PCC/PGL without a family history of the disease. Of the 62 patients, 40 (64.5%) had benign tumors, while 22 (35.5%) had malignant PCC/PGL, indicated by either lymph node or distant metastasis. Among the 40 patients with benign PCC/PGL, two (5.0%) were < 20 years old. The malignant PCC/PGL group consisted of 12 (54.5%) patients with PCC and 10 (45.5%) with PGL. The median PASS of the malignant PCC/PGL patients was > 4, significantly higher than that of benign PCC/PGL patients (*P* < 0.001). Malignant PCC/PGL tumors were larger than benign ones (*P* = 0.039). In addition, recurrence occurred in only 1/40 patient with benign PCC/PGL, with no deaths. Recurrence and death was observed in 14/22 (63.6%) and 4/22 malignant PCC/PGL patients (18.2%), respectively. Statistical analyses revealed no significant differences between benign and malignant PCC/PGL patients with regard to sex (*P* = 0.822), age (*P* = 0.535), disease pathology (*P* = 0.596) or follow-up duration (*P* = 0.125).

**Table 1:** Clinicopathologic characteristics of 12 PCC/PGL patients

No.	Sex	Age (year)	Type	Primary site	Metastasis site	Diagnosis	Size (cm)	Death	Recur
1	M	51	B	retroperitoneum		PGL	7	No	No
2	F	56	M	intraabdominal	LN	PGL	8	No	No
3	M	54	B	adrenal gland		PCC	11	No	No
4	M	51	B	retroperitoneum		PGL	6	No	No
5	M	52	M	retroperitoneum	bone	PGL	5.5	Yes	No
6	M	67	B	abdomen		PGL	7	No	No
7	F	41	B	retrocaval		PGL	5	No	Yes
8	M	58	M	bladder	bone, rectum	PGL	6.5	No	Yes
9	F	53	B	adrenal gland		PCC	9.5	No	No
10	M	70	B	retroperitoneum		PGL	7	No	No
11	F	40	B	retroperitoneum		PGL	7.5	No	No
12	M	47	M	retroperitoneum	LN	PGL	6	No	No

M, Male; F, Female; B, Benign; M, Malignant; PGL, paraganglioma; PCC, pheochromocytoma.

**Table 2:** Clinicopathologic demographics of patients with benign versus malignant PCC/PGL

Characteristics		Benign (*n* = 40)	Malignant (*n* = 22)	*p*-value
Sex	Male	23 (59.0%)	12 (52.2%)	0.822
	Female	17 (42.5%)	10 (45.5%)	
				
Age	≤ 20	2 (5.0%)	0 (0.0%)	0.535
	> 20	38 (95.0%)	22 (100.0%)	
				
Pathology	Pheochromocytoma	19 (47.5%)	12 (54.5%)	0.596
	Paraganglioma	21 (52.5%)	10 (45.5%)	
				
PASS	median (range)	2 (0–7)	5 (0–10)	< 0.001*
				
Tumor size (cm)	median (range)	5.0 (1.6–14.0)	6.8 (2.0–19.0)	0.039*
				
F/U duration (month)	median (range)	62.5 (0–170)	55.0 (5–254)	0.125*
				
Recurrent rate		1/40 (2.5%)	14/22 (63.6%)	< 0.01
				
Death rate		0/40 (0.0%)	4/22 (18.2%)	0.013

PASS, pheochromocytoma of the adrenal gland scaled score; *Mann-Whitney test.

### Genomic copy number alterations in benign and malignant PCC/PGL

**Figure 1:**
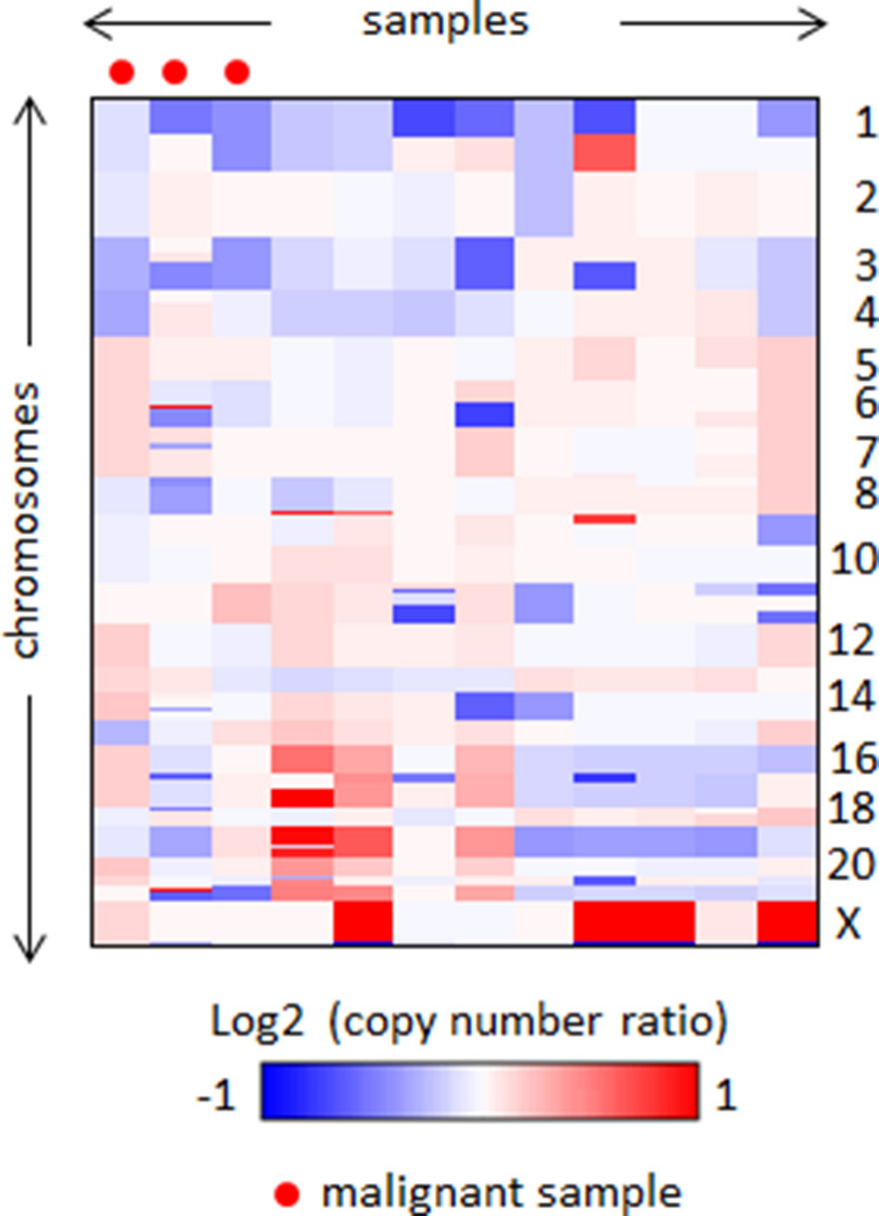
Heatmap of genomic profiles of the segmented copy number data

We did not observe any noteworthy focal amplifications or deletions via aCGH, and most samples showed few copy number aberrations regardless of malignancy status. Two regions, 1p and 3q, showed relatively frequent heterozygous loss in five and two cases, respectively (log ratio ≈ −0.5) (Figure 1). This indicates that copy number alteration is unlikely to be involved in PCC/PGL carcinogenesis, and other factors such as somatic mutations and gene fusions should be investigated to find relevant driver alterations. Additionally, there was no significant difference in genomic architecture between the malignant and benign samples.

### PNMT as a candidate marker for malignant PCC/PGL

To identify genes differentially expressed between benign and malignant tumors, we compared the mRNA expression profiles of three malignant and nine benign PCC/PGL specimens. Two hundred genes were overexpressed > 5-fold in malignant tumors. Upregulated genes were involved in either nervous system development (*MNX1, NRG1, PITX2, PAX2, FOXA1, POU3F2, TWIST1* and *NEUROG2*) or synaptic transmission (*BDNF, CACNB2, GAD1, NRG1* and *DRD4*) (Table S1). One hundred ninety genes were downregulated > 5 fold in the same tumors and were not associated with specific biological processes (Table S2). From the 390 differentially expressed genes, we selected phenylethanolamine N-methyltransferase (*PNMT*), an enzyme involved in the catecholamine biosynthesis pathway, as a candidate malignancy marker. *PNMT* presented the highest fold difference (benign/malignant fold change of ~160).

### Functional analysis of PNMT in PCC/PGL

**Figure 2:**
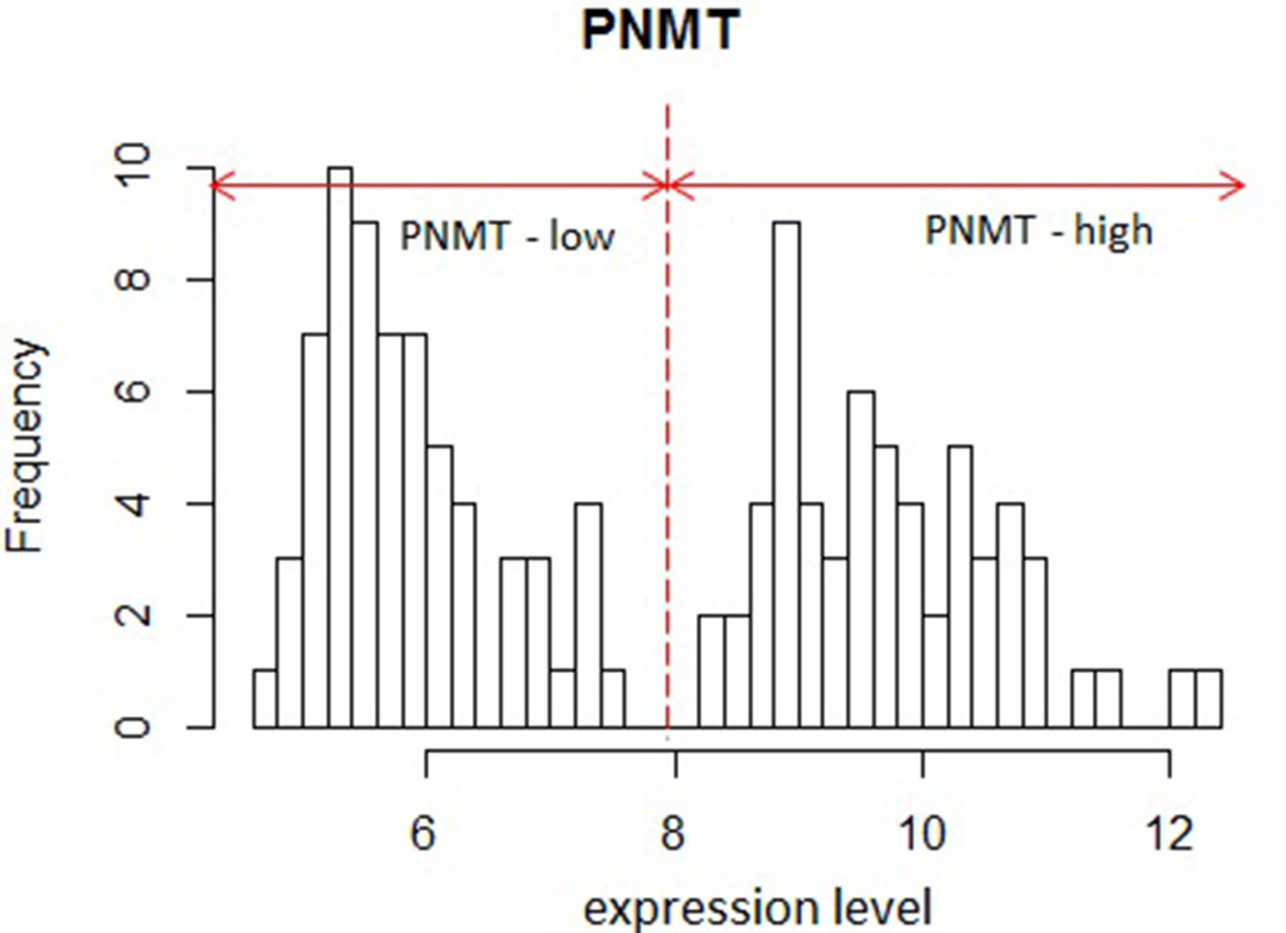
Histogram of PNMT expression in GSE19987 Two distinct distributions are shown.

**Figure 3:**
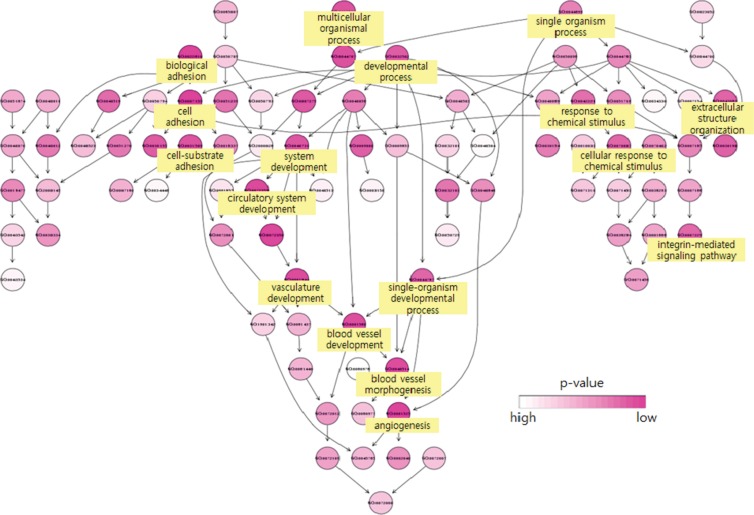
Biological functions of genes overexpressed in the *PNMT*-low group Overrepresentation of blood vessel development-related functions was observed upon analysis of the 200 highly upregulated genes in the *PNMT*-low group. Each circle represents a selected enriched gene ontology (GO) term, and darker color represents increased statistical significance.

To better characterize the function of *PNMT* in PCC/PGL, we analyzed a large (125 samples) public PCC/PGL microarray expression profile dataset (GSE19987) with a variety of mutations in pheochromocytoma susceptibility genes such as *RET*, *VHL*, *SDHB* and *SDHD* [22]. We performed functional analyses of genes differentially expressed in *PNMT*-high and -low groups. *PNMT*-high and -low groups were identified by their distinct normal distributions for *PNMT* expression in the dataset (Figure 2). About 200 highly upregulated genes [false discovery rate (FDR) < 0.0001] were identified in *PNMT*-low samples, including *TGFBI*, *VEGFA*, *CD34*, *CDH13*, *EFNA1*, *EPHB4*, *MFGE8*, *NOTCH1* and *PGF*. Gene Ontology (GO) analysis revealed overrepresentation of gene functions related to blood vessel development, morphogenesis and angiogenesis (Figure 3). We also identified approximately 650 overexpressed genes (FDR < 0.0001) in the *PNMT*-high group, which were mainly involved in synaptic transmission (*GATA3*, *NTGK1*, *TFAP2B* and *PHOX2A*), catecholamine metabolism (*PNMT*, *TH*, *GATA3*, *HPRT1* and *MAOB*) and Golgi vesicle transport (*VAMP2*, *CLTC*, *SCAMP1* and *SCFD1*)—all functions related to adrenalin hormone synthesis and secretion. These results suggest that low *PNMT* expression is related to aggressive PCC/PGL tumor development, and is supported by the fact that angiogenesis-related genes are upregulated in low *PNMT*-expressing tumors.

### Correlation of PNMT with hereditary PCC/PGL

**Figure 4:**
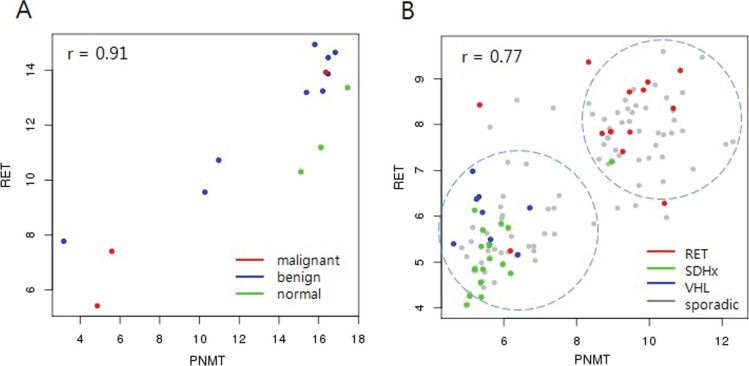
Correlation between *PNMT* and *RET* *RET* showed the highest correlation (*r* = 0.91) with *PNMT* in our data (**A**) Malignant tumors showed low expression of both *PNMT* and *RET*. *RET* showed the highest correlation (*r* = 0.77) with *PNMT* in the GSE19987 dataset, which includes hereditary PCC/PGL (**B**) Tumors harboring *RET* germline mutations showed high *PNMT* and *RET* expression, while *SDHx*/*VHL* germline-mutated tumors showed low expression.

In an analysis of the Pearson correlations between *PNMT* and all other genes in the microarray platform, the *RET* proto-oncogene showed the highest correlation with *PNMT* levels (0.91 and 0.77 in our dataset and GSE19987, respectively; Figure 4). *RET* is a well-known PCC/PGL susceptibility gene whose germ-line mutations are associated with hereditary disease. However, the correlation between *PNMT* and *RET* in this study was independent of *RET* mutation status (Figure 4A). Hereditary tumors harboring *RET* mutations overexpressed *PNMT*, while those harboring either *SDH* or *VHL* mutations downregulated *PNMT* (Figure 4B). This supports a previous study wherein unsupervised hierarchical cluster analysis of gene expression profiles of approximately 200 PCC/PGL samples separated hereditary tumors into two groups: *RET*/*NF1*- and *SDH*/*VHL*-related, with adrenergic and noradrenergic phenotypes, respectively [23]. The adrenergic/noradrenergic characteristics of PCC/PGL with *RET*, *SDH*, or *VHL* mutations largely reflect their origins from two types of chromaffin cells that can be distinguished based on *PNMT* expression. This provides a new platform to explore the pathogenic development of hereditary tumors from two different chromaffin cell populations.

### Validation of gene expression by quantitative IHC and qRT-PCR

**Figure 5:**
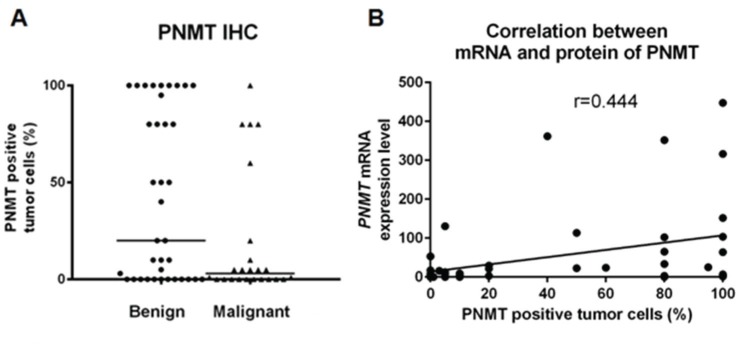
Comparison of benign and malignant PCC/PGL Comparative quantification of PNMT protein levels by immunohistochemistry (IHC) (**A**) Correlation between PNMT mRNA and protein levels (**B**).

**Figure 6:**
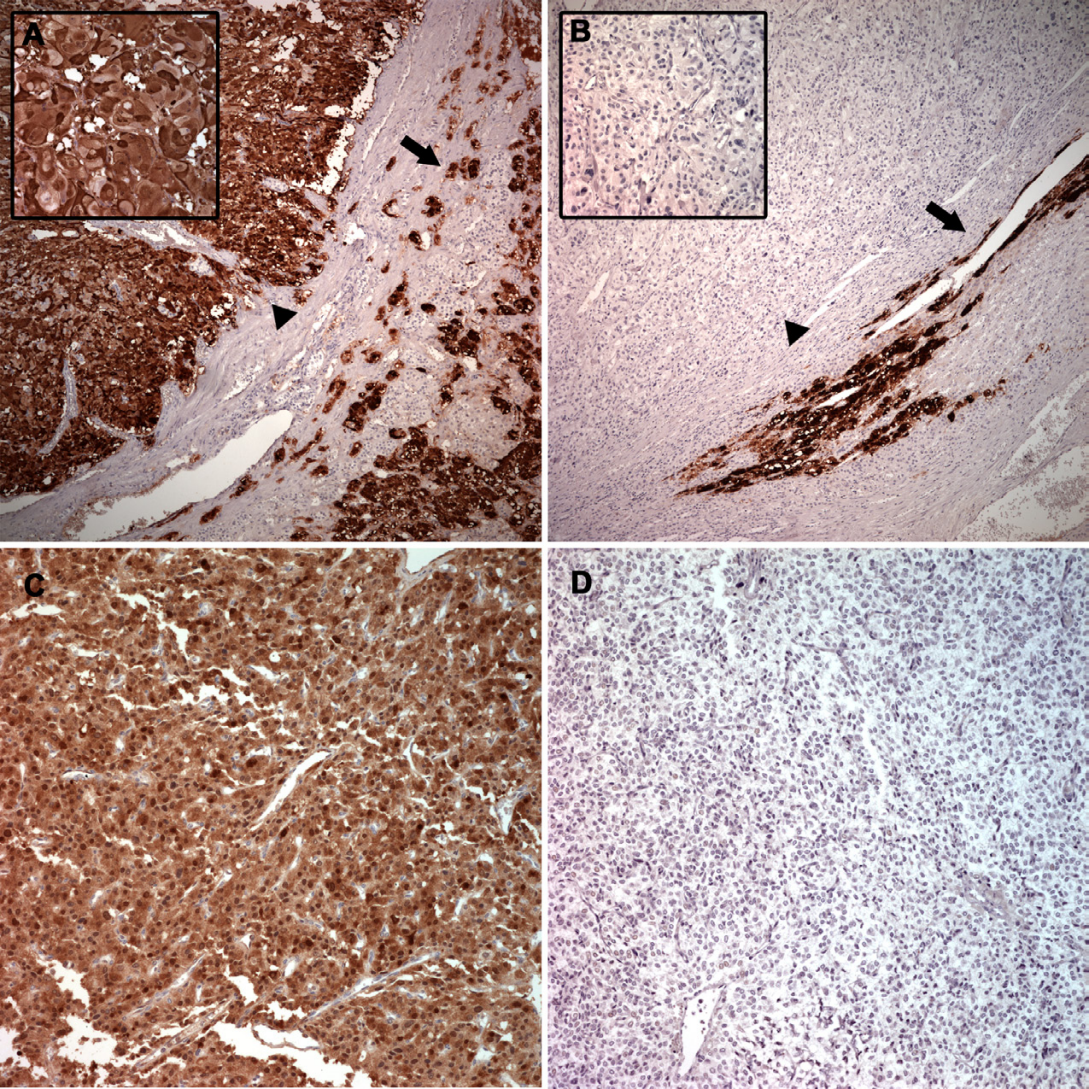
Representative PNMT protein levels in benign and malignant PCC and PGL Benign PCC (arrowhead) showed the same immunostaining intensity as the adjacent normal adrenal medulla (arrow) (**A**) Strong cytoplasmic staining was observed (left upper inset). Malignant PCC (arrowhead) exhibited complete absence of PNMT compared with the adjacent normal adrenal medulla (arrow) (**B**) Left upper inset represents higher magnification. Benign PGL showed diffuse and strong PNMT staining (**C**) Malignant PGL showed complete absence of PNMT positivity (**D**).

IHC analyses revealed PNMT staining predominantly within the cytoplasm, and the percentage of PNMT-positive cells was variable. PNMT staining was quantified by calculating the percentage of positively stained tumor cells, and was scored accordingly (Table 3). The median percentage of positive cells was 20% (range, 0–100) in benign PCC/PGL samples compared with 3% (range, 0–80) in malignant samples (*P* = 0.038; Figure 5A). In benign PCC/PGL, 46.2% of the samples presented > 50% positive cells, while 20.8% of the malignant samples stained > 50% for PNMT (*P* = 0.031, Figure 6). *PNMT* mRNA expression was successfully quantified by qRT-PCR in 52 of the 62 FFPE and 4 normal adrenal gland samples. The remaining cases failed to yield reliable qualities and/or quantities of RNA owing to the small size of tumor sections. We observed variable *PNMT* expression in normal adrenal gland tissues and benign and malignant PCC/PGL, ranging from 9.995–1610.673, 0.005–447.70 and 0.006–396.05, respectively, with median values of 48.3365, 8.55 and 3.44, respectively. *PNMT* expression was lower in benign than in malignant PCC/PGL, although this was not statistically significant (*P* = 0.069). PNMT mRNA and protein levels were correlated in PCC/PGL samples (*r* = 0.444, *P* = 0.001; Figure 5B) as well as in PCC (*P* = 0.076) and PGL samples (*P* = 0.133).

**Table 3:** PNMT immunohistochemical analysis in benign and malignant PCC/PGL samples were scored based on percentage of positive-staining cells

	Benign (*n* = 39^a^)	Malignant (*n* = 22)	
PNMT			*P*-value
0	12 (30.8%)	9 (40.9%)	0.031^b^
1+	2 (5.1%)	7 (31.8%)	
2+	7 (17.9%)	2 (9.1%)	
3+	18 (46.2%)	4 (18.2%)	

a The single remaining benign PCC/PGL case was not stained due to unavailability of a FFPE sample.

b Cochran-Armitage trend test.

### Correlation between PASS and PNMT immunoexpression

**Figure 7:**
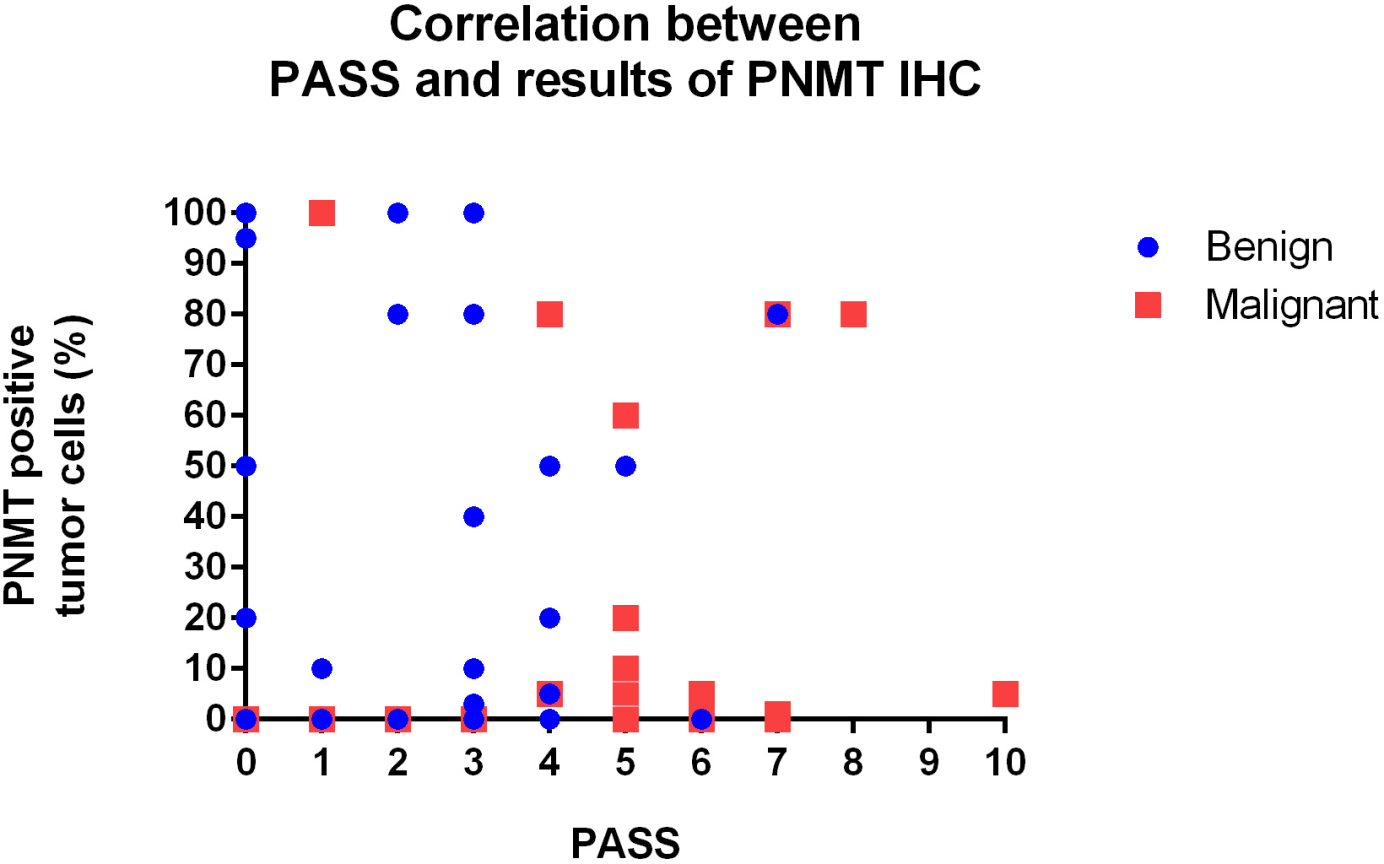
Correlation between PASS and PNMT protein levels

**Figure 8:**
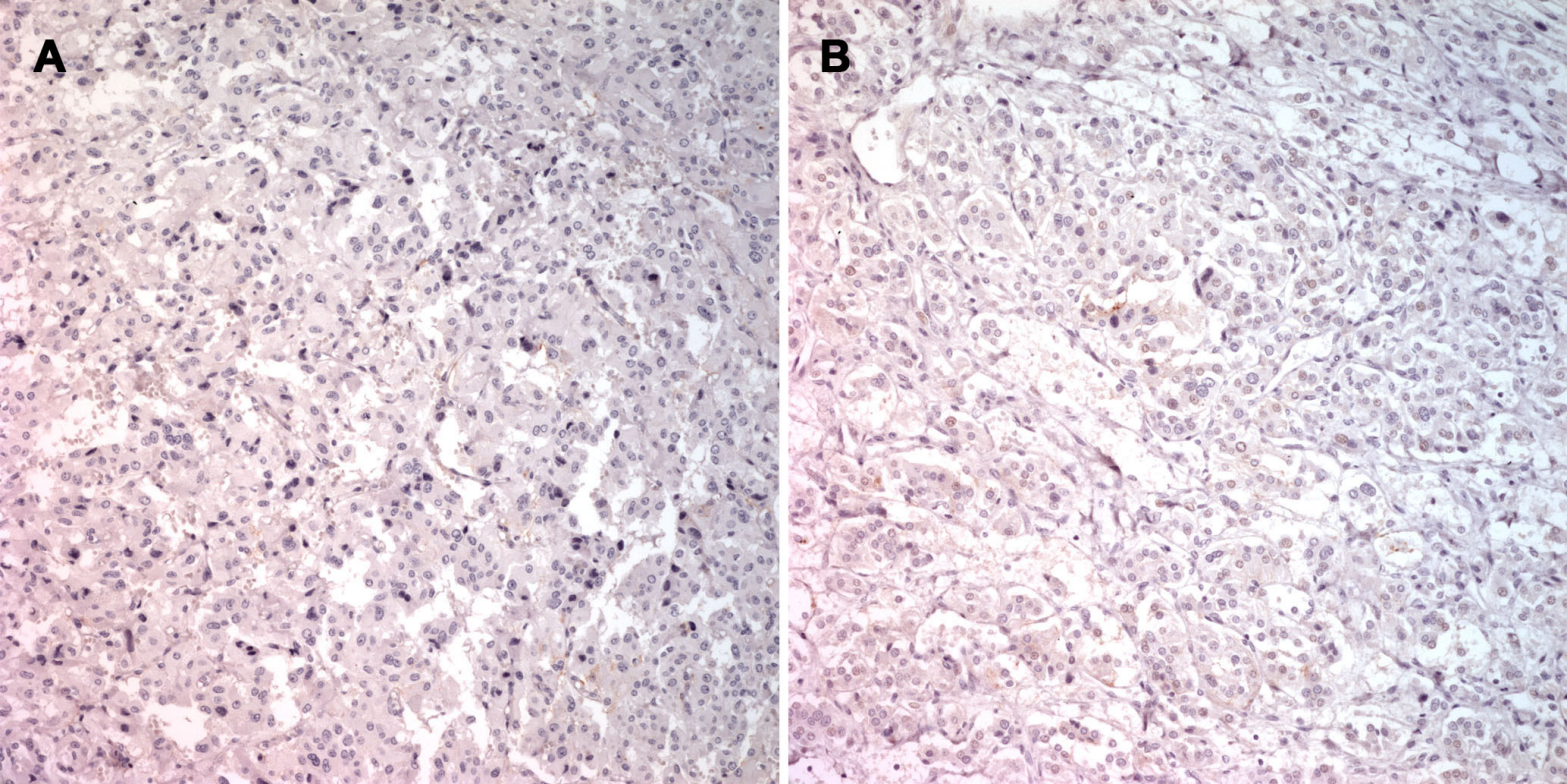
PNMT immunostaining in specimens from patient 7 Primary extra-adrenal pheochromocytoma in the right para-aortic area with complete absence of PNMT positivity (**A**) Recurrent extra-adrenal pheochromocytoma in the retrocaval space with complete absence of PNMT positivity (**B**).

A trend towards an inverse relationship between PASS and PNMT immunoexpression was observed, although this was not statistically significant (*r* = −0.140, *P* = 0.282; Figure 7). The case of patient number 7 was particularly interesting. Briefly, a 27-year old woman presented with hypertension and palpitation in March 1997. She had no family history of hypertension or any other known genetic disorder. After workup, she was diagnosed with extra-adrenal PCC in the right para-aortic area, which was treated by excision. In 2011, 14 years after the initial surgical excision, she once again presented with hypertension that lasted for three months. An abdominal and pelvis computerized tomography (CT) scan detected a 4.7 × 3.2 cm well-defined solid mass in the retrocaval space. The mass was excised and pathologically confirmed as recurrent extra-adrenal PCC. The initial PASS for this patient was < 4, suggesting a benign tumor. The PASS was 4 in the recurrent tumor, suggesting a potentially aggressive tumor. Although this patient relapsed, the recurrence site was the retroperitoneum, which did not meet criteria for malignancy. Therefore, this case was classified as benign for gene expression profiling in the current study. However, the gene expression profile of this patient was similar to those of malignant tumors. In addition, IHC revealed complete absence of PNMT protein expression in the primary and recurrent tumors (Figure 8), while qRT-PCR revealed low *PNMT* mRNA level, similar to malignant PCC/PGL. This finding suggests that the tumor might have a malignant genotype/phenotype and indicates limitations in the clinical and histopathological diagnosis of malignant PCC/PGL.

## DISCUSSION

In this study, we performed aCGH and gene expression profiling to identify candidate marker genes that might distinguish benign from malignant PCC/PGL. Microarray analysis revealed differential expression of 390 genes. We focused on *PNMT* for further investigation since it exhibited the highest fold change between benign and malignant samples. The PNMT enzyme plays a key role in adrenal medulla function, and its expression was downregulated in malignant PCC/PGL compared to benign tumors. We also performed functional analysis of genes that were differentially expressed in *PNMT*-high and -low expressing tumors. Approximately 200 highly upregulated genes (FDR < 0.0001) were identified in the *PNMT*-low samples. GO analysis revealed that these genes were commonly involved in angiogenesis.

The human *PNMT* gene is located on chromosome 17q21-q22 [24]. PNMT catalyzes the final step in the catecholamine biosynthesis pathway, in which norepinephrine is methylated to epinephrine [25, 26]. Although it is predominantly found in the chromaffin cells of the adrenal medulla, where a large proportion of the body's adrenaline is synthesized, PNMT activity has also been reported in various organs such as the heart [27, 28], kidney [29], lung, thymus and different parts of the brain [30]. *PNMT* gene expression was detected in the majority of these tissues [31–35].

There are few published studies on *PNMT* expression in PCC/PGL. Isobe, *et al.* [36] reported higher *PNMT* mRNA levels in normal adrenal medulla than in PCCs, which might explain why sporadic PCCs predominantly produce noradrenaline. The *PNMT* promoter contains glucocorticoid responsive elements (GREs) and *PNMT* transcription is modulated by glucocorticoids [37]. Therefore, elevated *PNMT* mRNA levels in normal adrenal medulla may be induced by stimulated glucocorticoid release from the adrenal cortex [38]. Low *PNMT* mRNA levels in PCCs might reflect an insufficient concentration of plasma cortisol in the artery supplying the tumor [36]. While it is generally believed that PNMT is absent in PGL, some investigators observed positive immunohistochemical PNMT staining in these tumors [39–41]. In our study, four cases of PGL in the head and neck showed loss of PNMT immunoexpression and extremely low *PNMT* mRNA levels. However, we did not observe any difference in PNMT protein and mRNA levels between PCC and PGL in our validation studies.

Eisenhofer, *et al.* reported that PCCs associated with multiple endocrine neoplasia type 2 (MEN 2) are adrenergic, whereas those associated with von Hippel-Lindau syndrome (VHL) are noradrenergic due to lower PNMT expression [42]. PNMT protein levels are reported to be significantly different between spontaneously occurring benign and locally invasive “malignant” PCC in mice [43]. Loss of PNMT regulatory mechanisms is characteristic of aggressive PCCs and is associated with a less mature phenotype, because PNMT is expressed after other catecholamine-synthesizing enzymes during embryogenesis [43]. This is consistent with our finding that over 80% of differentially expressed genes were downregulated in malignant tumors, and supports the hypothesis that malignant potential is related to a less differentiated expression pattern [43].

Although the association between PNMT and malignant PCC/PGL in humans was not previously reported, efforts have been made to find biochemical markers to predict malignancy [44]. Van der Hast, *et al.* reported that high dopamine, high norepinephrine and a high ratio of epinephrine to total catecholamine are associated with decreased metastasis-free survival [45]. Eisenhofer, *et al.* found that plasma methoxytyramine, the O-methylated metabolite of dopamine, was correlated with malignancy [46]. In our study, *PNMT* was downregulated in malignant PCC/PGL and PNMT protein expression differed between benign and malignant PCC/PGL. Although we did not evaluate plasma epinephrine or norepinephrine, our findings suggest that serum epinephrine is likely less abundant in patients with malignant PCC/PGL. Norepinephrine-producing tumors lacking PNMT are less differentiated than epinephrine-producing tumors. Accordingly, Kimura, *et al.* recently proposed a grading system for adrenal PCC/PGL (GAPP) that considers a tumor's biochemical phenotype and type of catecholamine produced when assessing metastasis risk [47].

Recently, PCC/PGL was divided into two main clusters based on tumor transcription profiles [48]. Cluster 1 was associated with hypoxia-related signals; cluster 2 with increased kinase signaling. Typically, patients with cluster 1 tumors present dopaminergic, noradrenergic, or a mix of dopaminergic and noradrenergic biochemical profiles. Those with cluster 2 tumors present either adrenergic or a mix of noradrenergic and adrenergic biochemical phenotypes. Tumors with noradrenergic profiles have an immature secretory phenotype, whereas those with either adrenergic or mixed noradrenergic/adrenergic profiles have a mature secretory phenotype. Metastasis risk is inversely correlated with maturity profile [48]. Thus, low PNMT expression and lack of epinephrine production might be associated with malignancy.

Use of PNMT as a diagnostic marker for malignancy could aid in the clinical management of PCC/PGL. IHC analysis of protein expression in FFPE samples is an effective and routine clinical diagnostic tool. Patients with clinically and histopathologically benign tumors at higher risk of malignancy based on low PNMT expression would benefit from frequent follow-ups, along with regular biochemical testing and imaging, to detect recurrent or persistent tumors. [48]. In light of the current practices, low PNMT expression with relation to catecholamine type might be associated with malignant behavior.

In conclusion, by combining aCGH with expression array analysis and validation studies, we identified *PNMT* as a candidate gene to distinguish malignant from benign PCC/PGL. This is the first demonstration of the association between *PNMT* downregulation and PCC/PGL malignancy in humans, and our findings identify *PNMT* as one of the most differentially expressed genes between malignant and benign tumors.

## MATERIALS AND METHODS

### Sample collection and study design

Fresh, frozen samples were obtained from 12 PCC/PGL patients according to a protocol approved by the Institutional Review Board between 2007 and 2013. Two pathologists studied hematoxylin/eosin-stained slides prepared from all frozen samples and confirmed that all samples contained tumor areas > 80%. All 12 samples were included in aCGH and expression microarray analyses. Normal adrenal gland medullas from three patients who underwent surgery for renal cell carcinoma were used as normal controls for expression microarray analysis.

To validate the results of altered gene expression and copy numbers analyses, 62 additional PCC/PGL FFPE samples were analyzed, including 19 malignant and 31 benign tumors. Eligibility criteria for including patients in the study were as follows: histological confirmation of PCC/PGL, availability of sufficient tissue for biomarker tests, and complete clinical and outcome information.

### Microarray analysis

Molecular profiling of the tumors was done using aCGH and expression microarray (Agilent Technologies, 60 K) following the manufacturer's protocol. Following segmentation using the GLAD algorithm, significantly amplified or deleted regions were identified by GISTIC (parameters: -ta 0.5 –td −0.5 –qvt 0.01). Genes differentially expressed in benign and malignant samples were selected based on their fold changes. Unsupervised hierarchical clustering was performed with a correlation metric to analyze similarity in expression among the 15 samples (12 pheochromocytoma, 3 normal adrenal medulla). The 390 genes showing the highest variance [interquartile range (IQR) > 4] across the 15 samples were selected for hierarchical clustering. To characterize *PNMT*, we analyzed a large public dataset (GSE19987; http://www.ncbi.nlm.nih.gov/gds/) that consisted of 125 pheochromocytomas with *RET*, *VHL*, and *SDHx* germline mutation data. *PNMT*-high and -low groups were defined according to the distribution of *PNMT* levels in the dataset. We used SAM (Significance Analysis of Microarrays) to identify genes that were differentially expressed between *PNMT*-high and -low groups. Biological functions related to *PNMT* were investigated by GO analysis using the R package GOstats (http://bioconductor.org/).

### RT-PCR analysis of *PNMT*

RNA was isolated from FFPE tumor samples using the RNeasy FFPE RNA Isolation Kit (Qiagen, Valencia, CA), and cDNA synthesis was performed using the SuperScript III first-strand kit (Invitrogen, Carlsbad, CA, USA) according to the manufacturers' instructions. The PCR primers and probes are described in Table S3. *HPRT1* and *GUSB* were used as endogenous control genes. RT-PCR analysis was performed with an ABI 7900HT Fast Real-time PCR system (Applied Biosystems, Foster City, CA, USA) using SYBR Green. Briefly, 1 μL of cDNA product was used as template in a 10 μL PCR reaction containing 5 μL of Power SYBR Green PCR master mix (Applied Biosystems) and 200 nM of each primer. All reactions were performed in triplicates. The amplification protocol was as follows: 95°C for 10 min; 40 cycles of 95°C for 10 s and 60°C for 60 s; and one cycle for melting curve analysis. The relative expression of *PNMT* compared with that of the reference genes was calculated using ΔCt (cycle threshold).

### IHC for PNMT

Tissues were fixed in 10% formalin solution, dehydrated through a graded ethanol series, cleared in xylene, and processed for embedding in paraffin wax according to routine protocol. The sections were placed in a 0.3% H_2_O_2_ solution for 15 min to inhibit endogenous peroxidase activity. They were then incubated with anti-PNMT primary antibody (1:400; ab119784, Abcam, Cambridge, UK) for 1 h at room temperature. The EnVision+ detection system for mouse antibodies (K4001, DAKO, Glostrup, Denmark) was used according to the manufacturer's instructions. Slides were then stained with liquid diaminobenzidine tetrahydrochloride (DAB+) using a high-sensitivity substrate-chromogen system (K3468, DAKO), and counterstained with Meyer's hematoxylin. Images were visualized with an Olympus BX40 light microscope (Olympus, Tokyo, Japan). Cytoplasmic staining was considered PNMT-positive, expressed as the percentage of positively stained tumor cells. The following criteria were used to score samples based on percent positive cells: 0, < 1%; 1, 1%–9%; 2, 10%–49%; and 3, > 50%.

### Statistical analyses

Either the χ^2^ or Fisher's exact test was used to examine associations between benign and malignant PCC/PGLs and their clinicopathological parameters. Differences in median PASS score, tumor size and follow-up duration between benign and malignant PCC/PGL were evaluated by the Mann-Whitney non-parametric test. *PNMT* mRNA level differences among normal adrenal tissue, benign PCC/PGL and malignant PCC/PGL was evaluated by the Kruskal-Wallis non-parametric test. Correlations between the percentage of PNMT protein immunopositive cells and PNMT mRNA level were investigated using the Spearman's rank correlation test. Correlations were evaluated using the Spearman's rank correlation coefficient. We used the Cochran-Armitage test to evaluate the PNMT immunoreactive score trend in benign and malignant PCC/PGL.

## SUPPLEMENTARY MATERIALS TABLES


