# Supplementary material for: Phenylethanolamine N-methyltransferase downregulation is associated with malignant pheochromocytoma/paraganglioma
Source: Oncotarget. 2016 Mar 21;7(17):24141–53. doi: 10.18632/oncotarget.8234 (PMC5029690; doi:10.18632/oncotarget.8234)
Supplement: Supplementary file 1 [file oncotarget-07-24141-s001.pdf]

Phenylethanolamine N-methyltransferase downregulation is associated with malignant pheochromocytoma/paraganglioma

Supplementary Materials

Supplementary Table S1: Upregulated genes expressed in malignant versus benign PCC/PGL

Gene symbol	Gene name	Benign average expression	Malignant average expression	Fold change
GAD1	glutamate decarboxylase 1 (brain, 67 kDa)	4.53	10.86	80.48
PITX1	paired-like homeodomain 1	4.46	10.53	67.16
CHRDL2	chordin-like 2	4.18	9.86	51.21
PCDH7	protocadherin 7	3.95	7.27	9.93
HMCN1	hemicentin 1	6.03	9.33	9.90
POU3F2	POU class 3 homeobox 2	6.15	9.18	8.12
PAX3	paired box 3	4.47	7.70	9.40
SATB2	SATB homeobox 2	5.32	8.59	9.66
WDR86	WD repeat domain 86	5.84	9.10	9.57
KCNG3	potassium voltage-gated channel, subfamily G, member 3	4.69	7.95	9.56
NRG3	neuregulin 3	5.79	8.54	6.70
FOXA1	forkhead box A1	4.71	7.19	5.59
FGFR2	fibroblast growth factor receptor 2	3.44	6.18	6.65

Supplementary Table S2: Downregulated genes expressed in malignant versus benign PCC/PGL

Gene symbol	Gene name	Benign average expression	Malignant average expression	Fold change
PNMT	phenylethanolamine N-methyltransferase	14.80	7.49	158.39
MYLK3	myosin light chain kinase 3	8.34	2.56	55.21
KCNT1	potassium channel, subfamily T, member 1	12.31	6.56	53.76
GHRH	growth hormone releasing hormone	11.29	5.56	52.98
VSTM2L	V-set and transmembrane domain containing 2 like	11.65	6.39	38.22
RGS22	regulator of G-protein signaling 22	9.04	4.08	31.01
ARC	activity-regulated cytoskeleton-associated protein	14.80	10.15	25.12
NEFH	neurofilament, heavy polypeptide	13.24	8.74	22.56
RET	ret proto-oncogene	13.08	8.63	21.78
KRT19	keratin 19	15.60	11.12	22.29

MGMT	O-6-methylguanine-DNA methyltransferase	12.69	9.85	7.14
GALNT6	UDP-N-acetyl-alpha-D-galactosamine:polypeptide N-acetylgalactosaminyltransferase 6 (GalNAc-T6)	11.97	8.28	12.86
KRT19P2	keratin 19 pseudogene 2	10.76	6.97	13.89
JAKMIP1	janus kinase and microtubule interacting protein 1	11.62	7.17	22.00
SLC32A1	solute carrier family 32 (GABA vesicular transporter), member 1	9.60	5.46	17.66

Supplementary Table S3: PNMT real-time PCR primers and probes

Gene	Sequence	Product size
PNMT-F	ATGATGTCAAGGGCGTCTTC	109 bp
PNMT-R	CCACTTCAAAGAACAGGGAATC	
HPRT1-F	TGACACTGGCAAAACAATGCA	94 bp
HPRT1-R	GGTCCTTTTCACCAGCAAGCT	
GUSB-F	CTCATTTGGAATTTTGCCGATT	81 bp
GUSB-R	CCGAGTGAAGATCCCCTTTTTA	
